# Influence of bone conduction transducer type and placement on ocular and cervical vestibular evoked myogenic potentials

**DOI:** 10.1038/s41598-021-87682-1

**Published:** 2021-04-19

**Authors:** Laura Fröhlich, Maira Wilke, Stefan K. Plontke, Torsten Rahne

**Affiliations:** Department of Otorhinolaryngology, Head and Neck Surgery, Martin Luther University Halle-Wittenberg, University Medicine Halle (Saale), Ernst-Grube-Str. 40, 06120 Halle (Saale), Germany

**Keywords:** Neurology, Medical research, Outcomes research

## Abstract

Evaluating the effectiveness of different bone conduction (BC) transducers with controlled coupling force to elicit cervical and ocular vestibular evoked myogenic potentials (cVEMPs, oVEMPs) in healthy subjects by comparing response rates, amplitudes, latencies, thresholds and asymmetry ratios. Prospective experimental study including healthy participants. VEMPs were measured to different stimulation modes; the BC transducer coupling force was controlled to 5.4 (± 0.5) Newton. cVEMPs: to bone conducted vibration (BCV) with the B81 transducer on the mastoid; oVEMPs: to BCV with the B81 on the mastoid, BCV with the B81 on the forehead, and BCV with the Mini-Shaker 4810 on the forehead. Air conducted sound (ACS) with insert earphones was used as reference. Data of 24 normal subjects (mean age 25.3 (± 3.0) years) were analyzed. ACS and BCV with the B81on the mastoid evoked cVEMPs in 100% of ears. The highest oVEMP response rates were obtained with the B81 on the mastoid (83–92%), the lowest with the B81 on the forehead (17–22%). The Mini-Shaker elicited lower response rates (65%) compared to results from the literature without coupling force control and compared to ACS (78–87%). Amplitudes were higher for BCV than ACS. ACS and BCV on the mastoid caused higher asymmetry compared to BCV forehead stimulation. The B81 was feasible to elicit VEMPs with mastoid placement and can be used as an approved medical device to measure BCV VEMPs in a clinical set-up. Normative asymmetry values have to be established due to higher variability for mastoid stimulation.

## Introduction

Cervical and ocular vestibular-evoked myogenic potentials (cVEMPs, oVEMPs) are routinely measured in many clinics as part of the neuro-otology test battery to evaluate otolith function. Cervical VEMPs were first described by Colebatch et al.^1^ who recorded click-evoked muscle reflexes from the ipsilateral sternocleidomastoid muscle. Ocular VEMPs were reported a decade later^2–4^. The underlying mechanism for VEMP recordings is that saccular and utricular afferents can be activated by air conducted sound (ACS) as well as by bone conducted vibration (BCV)^5^. The threshold of saccular afferents to ACS is approximately 15–20 dB lower than for utricular afferents^6^. Today it is known that due to differences in neural projections, utricular function can be assessed by recording the excitatory potentials from the contralateral ocular muscles (oVEMPs), while saccular function can be assessed by recording inhibitory potentials from the ipsilateral sternocleidomastoid muscle^7^. Clinically, cVEMPs are usually measured to ACS due to lower thresholds of saccular afferents to ACS and to avoid stretch reflexes, while BCV is normally used to measure oVEMPs^8^. Because of their vestibular origin, VEMPs can also be recorded in patients without hearing. However, the recording of VEMPs to ACS is not possible in patients with a conductive hearing loss. The impairment of sound transmission through the middle ear causes reduced VEMP amplitudes or absent responses^9,10^. Thus, BCV as an alternative stimulus has to be used to elicit the responses in these patients. Recordings in single neurons have shown that the threshold to BCV is lower than to ACS with reference to hearing levels^6,11^.

BCV stimuli can be generated by various types of bone conduction (BC) transducers. These include audiometric BC transducers, e.g., B71 or B81 (Radioear, New Eagle, USA), large vibratory shakers such as the Mini-Shaker 4810 (Brüel & Kjær Sound & Vibration Measurement A/S, Denmark) or tendon hammers.

The Mini-Shaker as a large electrodynamic vibratory shaker has been successfully used in numerous studies to measure oVEMPs and cVEMPs. It has a flat frequency response and sufficient maximum power output. However, the transducer is not specified as a medical device and is originally intended to be used for accelerometer calibration, vibration testing of small objects, and mechanical impedance and mobility measurements. Therefore, it is only applicable for experimental studies and not for routine clinical measurements. Furthermore, the set-up requires a specified power amplifier which is a costly and cumbersome set-up for clinical use. The shaker is very heavy with a weight of approximately 1 kg. It is usually hand-held during VEMP testing by the examiner and coupled to the test site by its weight, which corresponds to coupling forces between 10 and 20 N. The performance of the shaker when operated in the VEMP measurements was examined by Lütkenhöner^12^. It was found that the transducer’s properties strongly depend on the experimental conditions. The resonance frequency of the moving element changes as soon as the body of the shaker is held in the hand of an examiner. Moreover, the performance depends on the shaker’s spatial orientation. Most importantly, the static force by which it is pressed against a test person’s head can lead to fundamentally nonlinear behavior of the Mini-Shaker. This occurs if the force is too high so that the shaker’s table is statically displaced to its maximum position even if there is no electrical input. A set-up to control the shaker’s coupling force was described by Todd et al.^13^ who used the Mini-Shaker in a pulley system with a weight of 1–2 kg to maintain a constant force of 3.5–7 N while the transducer was coupled to the mastoid.

The use of tendon hammers to elicit VEMPs has also been described^14,15^. VEMPs could reliably be elicited by tendon hammer taps and the hammer itself is an approved medical device. However, for VEMP recordings, the tendon hammer has to be fitted with a pressure trigger linked to the recording system. The procedure allows assessment of whether a VEMP response is present or absent but manual tapping is difficult to control, lacks repeatability and is not calibrated with reference to force level making threshold measurements and comparisons difficult.

The audiometric BC transducer B71 has been used in a few studies, but the maximum output force generated by the transducer was not always sufficient to elicit VEMPs^14,16^. The newer B81 is a more powerful transducer based on the balanced electromagnetic separation transducer (BEST) principle which can produce higher output levels and less harmonic distortion than the B71^17^. The only study describing the use of the B81 to measure VEMPs was conducted by Mueller et al.^18^. Ocular VEMPs were measured in their study in a group of healthy subjects and the B81 was feasible to elicit the responses in all included subjects. The measurement of cVEMPs with the transducer has not been reported in the literature so far. The advantages of the B81 are that it is an approved medical device (CE marking) which is also used in audiometric evoked potential recordings (e.g., for BC auditory brainstem recordings) and can be easily calibrated to standard electrophysiological recording set-ups. The transducer is normally used with a steel spring headband and positioned on the mastoid so that a coupling force of 5.4 ± 0.5 N, as required for BC audiometry (ISO389-3^19^), is realized. The disadvantage of mastoid placement is that the responses are sensitive to small changes in stimulation site^20^ possibly making a comparison of the left and right side more difficult than for symmetrical otolith stimulation by forehead placement.

A new transducer, the B250, was recently described by Håkansson et al.^21^. It is based on the B81 design but slightly larger and optimized for output at 250 Hz. A first pilot study with healthy test subjects showed that cVEMPs and oVEMPs could successfully be elicited with the B250 positioned on the mastoid but not for forehead positioning. However, the transducer is not commercially available or approved as a medical device.

The objective of this study was to investigate the effectiveness of different BC transducers with controlled coupling force to elicit cVEMPs and oVEMPs in healthy subjects by comparing response rates, amplitudes, latencies, thresholds and asymmetry ratios. Cervical VEMPs to BCV were measured with the B81 on the mastoid, oVEMPs to BCV were measured with the B81 on the mastoid and forehead as well as with the Mini-Shaker on the forehead using an experimental apparatus to control the coupling force. As a reference, oVEMPs and cVEMPs to ACS were measured.

## Material and methods

### Study participants

The study was designed as a prospective comparative experimental study at a tertiary referral center. Based on the primary outcome measures, the study sample size was estimated to 25 participants. All test subjects had to be healthy adults and not older than 40 years to be included in the study. Screening audiograms were conducted in all subjects. Exclusion criteria were otologic or neurotologic disorders as well as an air–bone gap ≥ 10 dB at 500 Hz.

**Figure 1:**
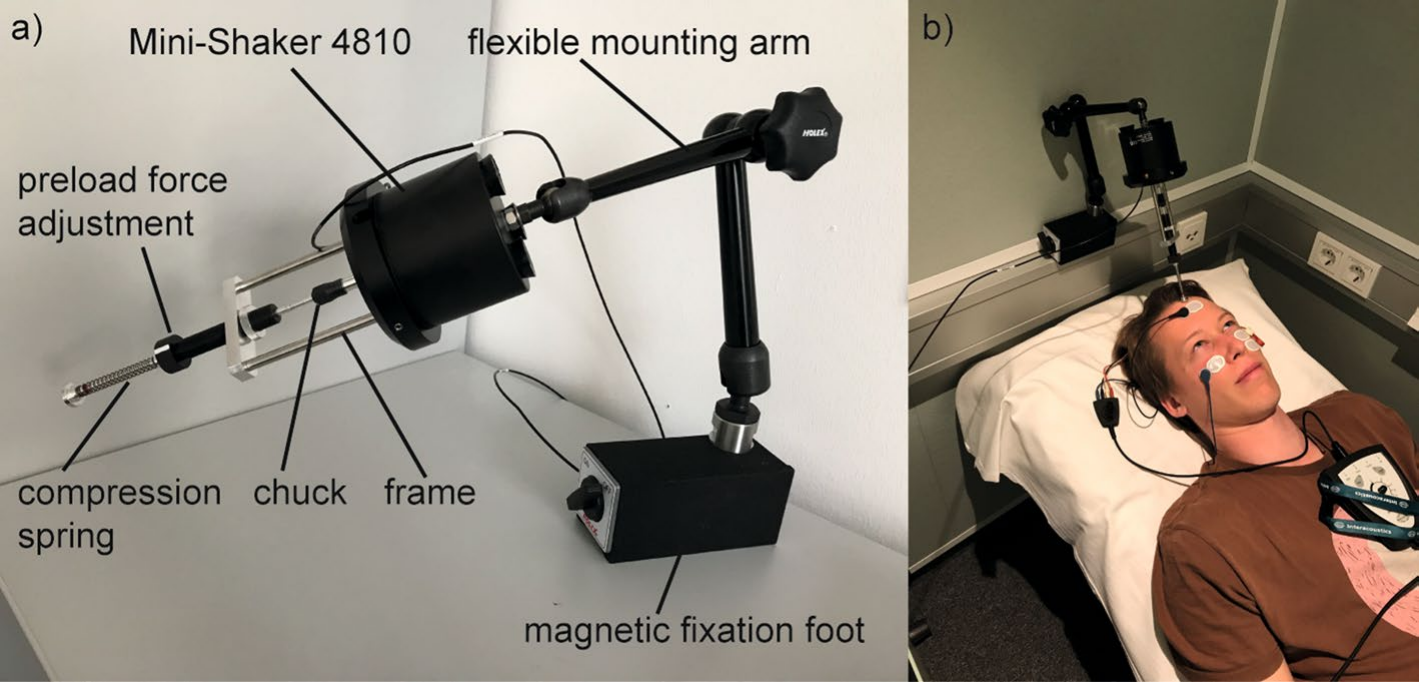
Experimental apparatus to control coupling force. (**a**) Mini-Shaker 4810 with the experimental apparatus to control the coupling force. (**b**) Mini-Shaker with experimental apparatus coupled to a test subject’s head at hairline for oVEMP testing. For publication of the image in an online open access publication, written informed consent was obtained by the pictured test person.

The study protocol was reviewed and approved by the ethics committee of the Medical Faculty of Martin Luther University Halle-Wittenberg and the University Hospital Halle (approval number: 2018-76). The study was performed in accordance with the ethical standards of the Declaration of Helsinki. Written informed consent was obtained from all participants before inclusion to this study. For publication of the image in Fig. 1 in an online open access publication, written informed consent was obtained by the pictured test person.

### Experimental set-up

The Eclipse EP25 system (Interacoustics A/S, Middelfart, Denmark) was used for stimulation and recording. Electromyographic (EMG) signals were recorded in a − 20 to 80 ms window relative to the onset of the stimulus and bandpass filtered between 10–1000 Hz. The artifact rejection level was set to 400 µV. Responses to at least 200 stimuli were averaged. Self-adhesive Neuroline 720 surface electrodes (Ambu A/S, Ballerup, Denmark) were used to record the EMG signals after the skin was prepared to provide impedances of less than 5 kΩ. The electrodes were placed over the middle of the sternocleidomastoid muscle ipsilateral to the stimulated side and referenced to an electrode over the sternum for cVEMP testing. For oVEMP recordings, the electrodes were placed as bipolar montage on the infra-orbital ridge 1 cm below the lower eyelid contralateral to the stimulated side and about 2 cm inferior to the first electrode.

For BCV stimulation, the B81 and the Mini-Shaker 4810 were used. The B81 was fitted with the standard P333 steel spring headband (Radioear, New Eagle, USA) to provide coupling forces of 5.4 ± 0.5 N. The Mini-Shaker was fitted with an experimental apparatus to control the coupling force (see Fig. 1). The apparatus consisted of a chuck holding an aluminum rod in a frame fixed to the shaker itself, and a preload force adjustment. The latter consisted of a compression spring with a spring constant of 0.49 N/mm. A static force of approximately 5 N was adjusted by screwing the preload force adjustment compressing the spring by 10 mm (10 mm × 0.5 N/mm = 5 N). The aluminum rod ended with a plane circular tip surface of approximately 1.75 mm^2^ which served as an adapter to be coupled to the hairline at the test person’s head. The whole system was fixed to a flexible mounting arm. The Mini-Shaker was used with the power amplifier 2718 (Brüel & Kjær Sound & Vibration Measurement A/S, Denmark). For ACS stimulation, ER-3A insert earphones (3 M, St. Paul, MS, USA) were used.

The stimuli were short 500 Hz tone bursts (0–1–0, i.e.: 0 cycles rise/fall time, 1 cycle plateau) for each measurement. The BCV stimuli were calibrated on an artificial mastoid 4930 (Brüel & Kjær Sound & Vibration Measurement A/S, Denmark). An InfiniiVision 2000 X-Series oscilloscope (Keysight Technologies, Santa Rosa, CL, USA) was used to read the output peak-to-peak equivalent vibratory force levels (peVFL). The maximum stimulus intensity was 142 dB peVFL for the B81 and 144 dB peVFL for the Mini-Shaker. For ACS, the standard clinical units and calibration in dB nHL was used. The maximum stimulus intensity was 100 dB nHL. The stimulus polarity was chosen to produce outward initial movement, i.e., a force towards the head, for all transducers and measurement modes. The B81 is polarized when manufactured to give the same direction of movement for a given voltage polarity. The Mini-Shaker’s polarity varies for every device so that the polarity for the specific transducer used in this study was controlled using the artificial mastoid and the oscilloscope to ensure the correct voltage polarity corresponsing to outward initial movement was chosen in the Eclipse set-up menu.

### Procedures

Ocular and cervical VEMPs were measured on the right and left sides of all subjects.

#### cVEMPs

For cVEMP recordings, the participants were sitting on a chair and were asked to turn their head to the shoulder contralateral to the stimulated side^22^. VEMP responses were recorded from the ipsilateral sternocleidomastoid muscle. The EMG signal was monitored, and feedback was provided by the examiner to ensure sufficient tonic muscle contraction (50–200 µV). To calculate the background EMG, the root mean square of the EMG signal was averaged over the pre-stimulus window and for each recording frame. EMG scaling was performed by normalizing the cVEMP p13-n23 peak-to-peak amplitude to the background EMG in order to reduce the impact of muscle contraction on cVEMP amplitudes.

Cervical VEMPs were measured to BCV using the B81 positioned on the mastoid and to ACS using the ER-3A insert earphones. The Mini-Shaker was not used for cVEMP measurements because the experimental apparatus required a fixed head position and could not be coupled to the head during cVEMP measurements (turning the head to the contralateral shoulder was necessary).

#### oVEMPs

For oVEMP testing, the participants were asked to lie in supine position and look up, maintaining an upward gaze of approximately 30°. Upward gaze was controlled by asking the participants to looking at a certain point on the ceiling serving as a target. VEMP responses were recorded from the ocular muscles contralateral to the stimulated side.

Ocular VEMPs were measured to BCV using the B81 on the mastoid and on the forehead as well as using the Mini-Shaker on the forehead. During the measurements with the Mini-Shaker the test person’s head was positioned on a fixation pillow (mediPlac, Borchen, Germany) to prevent head tilt and therefore prevent a change of coupling force adjusted with the experimental apparatus described earlier. Ocular VEMPs to ACS were measured using the ER-3A insert earphones.

### Data analysis

The VEMP responses were analyzed in OtoAccess software version 1.5 (Interacoustics A/S, Middelfart, Denmark, https://www.interacoustics.com/otoaccess/database). A response was judged to be present when it was clearly larger than the pre-stimulus waveforms, i.e., the background noise. The amplitudes of all myogenic potentials were measured from peak to peak (p13-n23 for cVEMPs, n10-p15 for oVEMPs). The asymmetry ratio (AR) in % was calculated by dividing the difference between the larger and smaller amplitude by the sum of the amplitudes multiplied by 100.

Descriptive statistics were used to report latency, amplitude, threshold and asymmetry data. Quantitative data were presented as mean, standard deviation (±), and range (minimum and maximum).

Response rates were calculated for each stimulation mode (cVEMP: B81 at mastoid, AC; oVEMP: B81 at mastoid, B81 at forehead, Mini-Shaker at forehead, AC) for the right and left sides and were compared using the Chi-square test. For confirmed associations (*p* < 0.05), post-hoc analysis was performed by pairwise Chi-square tests. To consider multiple comparisons, the *p* value was reduced according to the Bonferroni correction (cVEMPs: 4 stimulation modes with six pairwise comparisons, *p* = 0.05/6 = 0.008). Qualitative data were presented as graphs, if appropriate. SPSS version 25 for Windows software (IBM, Armonk, NY, USA, https://www.ibm.com/products/spss-statistics) was used for all statistical analyses. Graphs were created in GraphPad Prism version 8.4.3 (Graphpad Software, San Diego, CA, USA, https://www.graphpad.com/scientific-software/prism/).

## Results

Twenty-four subjects could be included into the study. The mean age was 25.3 (± 3.0) years (range 19–30 years). Twelve participants were male, 12 were female. Due to technical problems with the measurement set-up during two data collection sessions, the AC oVEMP and BC oVEMP data with B81 on the forehead and the Mini-Shaker from another subject were lost. Thus, cVEMP data were available from 24 subjects and 48 ears, and oVEMP data were available from 23 subjects and 46 ears except for BC oVEMPs with the B81 on the mastoid (24 subjects and 48 ears).

**Figure 2:**
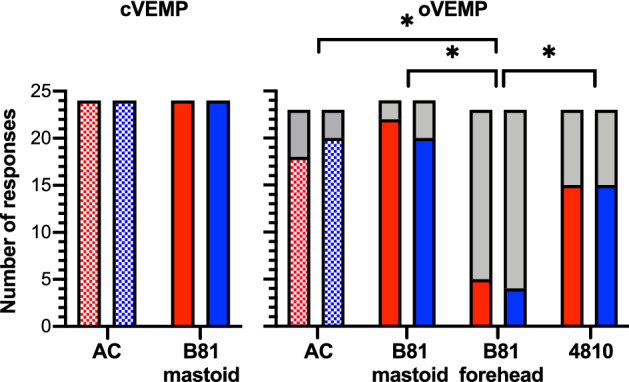
VEMP response rates. oVEMP and cVEMP response rates as absolute numbers for the left (blue) and right (red) side and different (bone conduction) transducer types and placements. Absent responses are illustrated in gray. Chi-square tests showed that the oVEMP response rate with the B81 on the forehead was lower compared to all other stimulation modes (*p* < 0.008, marked by asterisks).

The cVEMP and oVEMP response rates are illustrated in Fig. 2 for the different stimulation modes. Cervical VEMPs were present to BCV with the B81 on the mastoid as well as to ACS in all 24 subjects for the left and right sides (100%). Ocular VEMPs were obtained with the B81 on the mastoid in 22 of 24 right ears (92%) and 20 of 24 left ears (83%). Bilateral absence of responses was observed in 2 subjects, and unilateral absence on the left side in 2 subjects. When the B81 was positioned on the forehead, 5 of 23 right ears (22%) and 4 of 23 left ears (17%) showed oVEMP responses. The oVEMPs were absent bilaterally in 18 subjects and unilaterally on the right side in 1 subject. With the Mini-Shaker, oVEMPs were measurable in 15 of 23 right and left ears (65%). In the other 8 subjects, the oVEMPs were absent bilaterally. To ACS, oVEMPs were obtained in 18 of 23 right ears (78%) and 20 of 23 left ears (87%). In 3 subjects, responses were absent bilaterally and in 2 subjects, they were absent on the right side only.

The Chi-square test showed an association between the oVEMP response rate and stimulation mode for the right ears (*Χ*^*2*^(3) = 28.02, *p* = 0.000) and left ears ((*Χ*^*2*^(3) = 30.34, *p* = 0.000). The post-hoc pairwise Chi-square tests showed that the B81 on the forehead was different with respect to response rate compared to all other stimulation modes (*p* < 0.008 for all comparisons). No other differences in the response rates were observed between the other stimulation modes.

**Figure 3:**
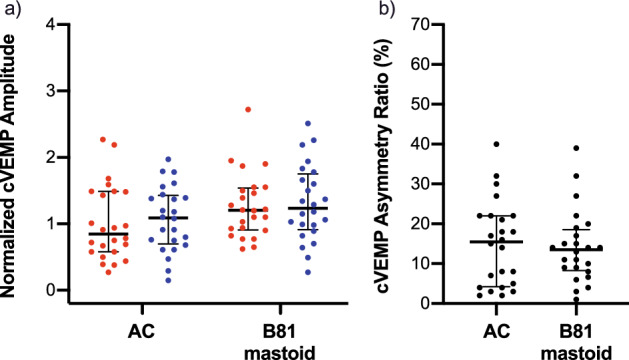
cVEMP amplitudes and asymmetry ratios. (**a**) cVEMP normalized amplitudes and (**b**) asymmetry ratios with medians and interquartile ranges for the left (blue) and right (red) side and different (bone conduction) transducer types and placements.

The means and standard deviations of the latencies, amplitudes, thresholds and ARs are summarized in Table 1 (cVEMPs) and Table 2 (oVEMPs) for the different stimulation modes and the left and right sides. For cVEMPs, there were no clinically relevant differences between the latencies for BCV and ACS. The mean p13 latencies were between 14.1 (± 1.4) and 15.6 (± 2.0) ms and n23 latencies were between 23.5 (± 2.4) and 24.6 (± 2.0) ms. The mean normalized cVEMP amplitudes were larger by approximately 0.3 for BCV compared to ACS, i.e., in a clinically relevant range. The mean cVEMP ARs were not different between BCV (14.2 (± 9.0)%) and ACS (15.0 (± 10.7)%). Figure 3 shows the amplitude and AR data distributions including medians and interquartile ranges.

**Table 1:** Means and standard deviations ( ±) of cVEMP latencies, amplitudes, thresholds, and asymmetry ratios for the left and right side and different (bone conduction) transducer types and placements.

Stimulation mode	p13 (ms)		n23 (ms)		Amplitude (normalized)		Threshold (dB)^a^		Asymmetry ratio (%)
cVEMPs	R	L	R	L	R	L	R	L	
AC	14.1 ± 1.4	15.2 ± 1.9	23.5 ± 2.4	23.7 ± 2.4	1.0 ± 0.6	1.1 ± 0.5	91.0 ± 6.1	90.0 ± 5.5	15.0 ± 10.7
B81 @ Mastoid	14.5 ± 1.6	15.6 ± 2.0	24.6 ± 2.0	24.5 ± 2.3	1.3 ± 0.5	1.3 ± 0.6	127.0 ± 4.4	128.5 ± 4.3	14.2 ± 9.0

AC: air conduction; R: right; L: left; ^a^Threshold in dB peVFL for bone conduction and dB nHL for air conduction.

**Table 2:** Means and standard deviations ( ±) of oVEMP latencies, amplitudes, thresholds, and asymmetry ratios for the left and right side and different (bone conduction) transducer types and placements.

Stimulation mode	n10 (ms)		p15 (ms)		Amplitude (µV)		Threshold (dB)^a^		Asymmetry ratio (%)
oVEMPs	R	L	R	L	R	L	R	L	
AC	9.3 ± 0.5	9.6 ± 1.1	14.0 ± 1.0	14.5 ± 1.5	4.5 ± 2.3	3.8 ± 2.4	94.7 ± 4.7	94.8 ± 4.3	22.2 ± 15.8
B81 @ Mastoid	9.5 ± 1.0	10.1 ± 2.1	14.6 ± 1.0	15.1 ± 2.0	5.8 ± 3.7	5.9 ± 2.9	130.0 ± 4.9	130.5 ± 4.9	23.1 ± 18.7
B81 @ Forehead	12.0 ± 0.7	11.4 ± 0.7	16.6 ± 0.2	15.7 ± 0.7	1.5 ± 0.7	1.8 ± 0.3	139.0 ± 4.5	138.3 ± 4.8	10.3 ± 7.5
4810 @ Forehead	8.6 ± 0.5	8.9 ± 0.7	12.9 ± 1.1	13.1 ± 1.0	5.4 ± 3.6	5.2 ± 4.7	140.7 ± 2.4	141.0 ± 2.5	14.9 ± 13.4

AC: air conduction; R: right; L: left; ^a^Threshold in dB peVFL for bone conduction and dB nHL for air conduction.

**Figure 4:**
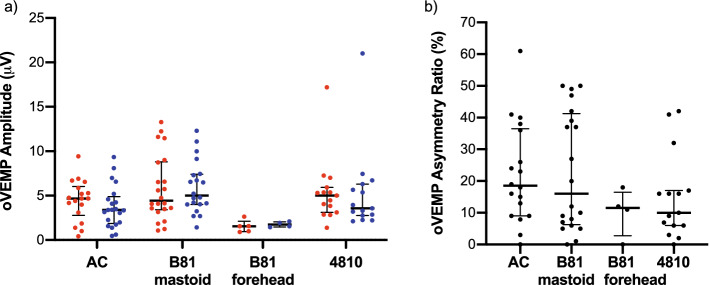
oVEMP amplitudes and asymmetry ratios. (**a**) oVEMP amplitudes and (**b**) asymmetry ratios with medians and interquartile ranges for the left (blue) and right (red) side and different (bone conduction) transducer types and placements.

In oVEMP recordings, the B81 on the forehead resulted in the longest n10 (12.0 (± 0.7) ms for right ears) and p15 latencies (16.6 (± 0.2) ms for right ears). The shortest latencies were obtained when the Mini-Shaker was used (n10: 8.6 (± 0.5) ms for right ears; p15: 12.9 (± 1.1) ms for right ears). The mean n10-p15 interpeak latencies were between 4.1 (± 0.8) ms (Mini-Shaker, left ears) and 5.0 (± 0.7) ms (B81 on the mastoid, right ears), i.e., not different between the different stimulation modes. As for cVEMPs, the mean oVEMP amplitudes were larger for BCV compared to ACS by 0.7–2.1 µV except for the B81 on the forehead which produced the smallest amplitudes. The ARs were similar for the B81 on the mastoid (23.1 (± 18.7)%) and ACS (22.2 (± 15.8)%) but lower for the Mini-Shaker (14.9 (± 13.4)%) and the B81 on the forehead (10.3 (± 7.5)%). Figure 4 shows the amplitude and AR data distributions including medians and interquartile ranges. Forehead stimulation with the B81 or Mini-Shaker required approximately 10 dB higher output levels to reach the oVEMP thresholds compared to stimulation at the mastoid. The mean oVEMP thresholds were up to 139.0 (± 4.5) dB peVFL for the B81 on the forehead and 141.0 (± 2.5) dB peVFL for the Mini-Shaker compared to 130.5 (± 4.9) dB peVFL with the B81 on the mastoid.

## Discussion

This is the first study measuring BC cVEMPs with the B81 BC transducer, showing the feasibility of the device. This is also the first study comparing oVEMPs measured with the B81 to oVEMPs measured using a Mini-Shaker with controlled coupling force.

The feasibility of using the audiometric BC transducer B81 on the mastoid to measure cVEMPs and oVEMPs could be confirmed in a young and healthy study sample. The cVEMP response rate was 100%. The oVEMP response rate with the B81 on the mastoid was comparable to using the Mini-Shaker with controlled coupling force. However, controlling the coupling force of the Mini-Shaker resulted in lower response rates compared to data from the literature, where the transducer was pressed against the forehead or mastoid by its own weight^16,23^.

There is the risk that the experimental apparatus introduced in this study was not feasible to produce the desired coupling force, possibly due to small head movements by the test person. The apparatus requires a stable head position which was optimized during the measurement by using a fixation pillow. However, small head movements cannot be excluded so that the final peak-to-peak output vibratory force level might have been too small to elicit oVEMP responses. We proposed that it could be beneficial for VEMP testing, if the Mini-Shaker’s coupling force was controlled to avoid nonlinear operation modes. However, the response rates showed that the nonlinear operation when pressed against the forehead by the shaker’s own weight is advantageous for VEMP measurements.

Forehead stimulation required approximately 10 dB higher stimulus intensities than mastoid stimulation to evoke oVEMPs. Thus, small changes in coupling force and therefore in stimulus intensities are very likely to have caused absent oVEMPs to stimulation by the Mini-Shaker in some cases. The need for higher output levels for forehead stimulation to elicit VEMPs also explains why the response rates to forehead stimulation with the B81 were clearly lower compared to the other stimulation modes. The maximum output levels generated by the B81 were slightly lower than with the Mini-Shaker and most likely not feasible to elicit oVEMPs in most test subjects. It has been shown in a few studies that oVEMPs are very sensitive to direction of skull acceleration so that variation of stimulus site along the midline of the skull results in change of polarity and/or latency^24–26^.

Beside the effect of stimulus site on the VEMP response polarity it is also known that the BCV stimulation polarity (inward versus outward initial transducer movement) can influence the VEMP response, especially latencies^27^. Thus, it is important to consider the stimulation polarity when interpreting or comparing the results of this study to others. Initial force towards the head was chosen in this study for all measurements. This could explain the small but noticeable latency difference observed between forehead and mastoid stimulation.

The results of this study showed that amplitudes to BCV were higher than to ACS for oVEMPs and cVEMPs. This has been reported in other studies before^18,23,28^. When VEMP amplitudes are small, BCV could be used to decide whether a response is present or absent. Thus, the amplitude differences are relevant for clinical decision making.

It was also observed in this study that ARs of oVEMP amplitudes were larger for the B81 on the mastoid and for ACS compared to transducer forehead placement. The absolute ARs with the Mini-Shaker on the forehead and with the B81 on the mastoid were comparable to values reported in the literature for these stimulation modes^14,18^. The AR difference between mastoid and forehead placement has been described before for the Mini-Shaker^16^. Higher ARs for lateral acceleration are possibly caused by variations in transducer placement on the left and right mastoids resulting in small differences of stimulus intensities at the otolithic receptors. It has been shown that VEMP responses to lateral acceleration can be sensitive to small variations in transducer placement^20^. Thus, the acquisition of normal data is very important for clinical set-ups using mastoid placement in order to define cut-off ARs to identify pathologic VEMP results. Forehead placement is advantageous due to symmetrical stimulation of both labyrinths but cannot be realized using B81 as reported earlier.

In a study by Rosengren et al.^16^ it was observed that ACS stimulation and BCV tone bursts delivered to the mastoid were less effective in evoking oVEMPs than cVEMPs. They also reported that the responses had high degrees of asymmetry. Forehead taps by a tendon hammer and lateral pulses produced more symmetrical responses and had comparable response rates in their study. These findings were confirmed in our study. However, despite the drawback of higher asymmetry, the B81 was shown to be a feasible transducer and most important an approved medical device to elicit BCV oVEMPs and cVEMPs when placed on the mastoid. Due to lower thresholds for mastoid stimulation compared to stimulation on the forehead, the output generated by the B81 audiometric BC transducer was sufficient for VEMP measurements. To overcome the drawback with high asymmetry, a more powerful transducer needs to be developed as medical device which is sufficient to elicit VEMPs when positioned on the forehead. The approach to use pulses instead of tone bursts as described by Rosengren et al. is not applicable for the B81. Pulses would lead to further oscillations due to the transducer’s technical design as a variable reluctance type transducer. The B81 was designed as an audiometric transducer to be operated within a relatively narrow frequency range. While this gives the advantage of being an approved medical device, the mechanical design is not feasible for impulsive stimulation which is very effective for utricular stimulation.

## Conclusion

The B81 proved to be a feasible BC transducer to elicit oVEMPs and cVEMPs when placed on the mastoid. As an approved medical device, the B81 is a suitable transducer for clinical VEMP measurement set-ups. Due to variability in placement on the mastoids and resulting larger ranges of asymmetry ratios, normative data have to be collected for every set-up to determine cut-off asymmetry ratios for identifying pathologic results. A more powerful and approved transducer would be beneficial, sufficient to elicit VEMPs to forehead stimulation.

In future studies, VEMPs measured with the B81 should also be investigated in a representative study sample including older test subjects as well as in patients with pathologies (e.g., third window pathologies, vestibular schwannoma, cochlear implant users).

## Data Availability

The datasets generated for this study are available on request to the corresponding author.
